# Basosquamous Carcinoma: A Commentary

**DOI:** 10.3390/cancers13236146

**Published:** 2021-12-06

**Authors:** Christina Fotiadou, Zoe Apalla, Elizabeth Lazaridou

**Affiliations:** Second Department of Dermatology—Venereology, Aristotle University, Medical School, 54124 Thessaloniki, Greece; zapalla@auth.gr (Z.A.); bethlaz@auth.gr (E.L.)

**Keywords:** basosquamous carcinoma, metatypical basal cell carcinoma, diagnosis, treatment, biologic behavior, dermoscopy, histopathology, Mohs’ micrographic surgery, genetics, vismodegib

## Abstract

**Simple Summary:**

Basosquamous carcinoma is a rare, aggressive non-melanoma skin cancer with features that lie between those of basal cell carcinoma and squamous cell carcinoma. A lot of controversy has been raised around the classification, pathogenesis, histologic morphology, biologic behavior, prognosis and management of this tumor. This is a narrative review based on articles published on PubMed in English language which had in their title the terms “basosquamous carcinoma” and/or “metatypical carcinoma of the skin”. The aim of this review was to summarize and evaluate the latest data of the English literature regarding epidemiology, clinical presentation, dermoscopic and histopathologic characteristics, as well as the genetics and management of BSC to better characterize basosquamous skin lesions.

**Abstract:**

Basosquamous carcinoma is a rare, aggressive non-melanoma skin cancer with features that lie between those of basal cell carcinoma and squamous cell carcinoma. A lot of controversy has been raised around the classification, pathogenesis, histologic morphology, biologic behavior, prognosis and management of this tumor. This is a narrative review based on an electronic search of articles published in PubMed in English language which had in their title the terms “basosquamous carcinoma” and/or “metatypical carcinoma of the skin”. The aim of this review was to summarize and evaluate current data regarding epidemiology, clinical presentation, dermoscopic and histopathologic characteristics, as well as the genetics and management of BSC, in order to shed some more light onto this intriguing entity. As a conclusion, dermoscopy, deep incisional biopsies and immunohistologic techniques (Ber-EP4) should be applied in clinically suspicious lesions in order to achieve an early diagnosis and better prognosis of this tumor. Surgical treatments, including wide excision and Mohs’ micrographic surgery, remain the treatment of choice. Finally, vismodegib, a Hedgehog pathway inhibitor, must be thoroughly investigated, with large controlled trials, since it may offer an alternative solution to irresectable or difficult-to-treat, locally advanced cases of basosquamous carcinoma.

## 1. Introduction

Basosquamous carcinoma (BSC) is a rare, relatively aggressive non-melanoma skin tumor which has raised a lot of controversy regarding its classification, its pathogenesis and its management since it was first described in the early 20th century. Multiple diagnostic and treatment challenges arise from the fact that BSC has a variable and non-characteristic clinical and histologic morphology seated between that of basal cell carcinoma (BCC) and squamous cell carcinoma (SCC) that is followed by a rather unpredictable biologic behavior.

This article aimed to summarize and evaluate all the latest data of the English language literature regarding epidemiology, clinical presentation, dermoscopic and histopathologic characteristics, as well as the genetics and management of BSC, in order to shed some more light to this intriguing entity.

## 2. Materials and Methods

A PubMed search of articles in English was conducted using the term BSC and/or metatypical BCC alone or with the following subheadings: classification, incidence, epidemiology, diagnosis, histopathology, dermoscopy, genetics, biologic behavior, treatment.

Inclusion criteria were: (1) case series or case reports of BSC; and (2) review articles, meta-analyses and systematic reviews on BSC;

Exclusion criteria were: (1) articles in a language other than English (i.e., French, German, Spanish, Chinese, etc.); and (2) articles about BSC of organs other than the skin (i.e., larynx, nasopharynx, lungs, anus, etc.);

Additionally, selected articles that were referenced in the included publications were used to support the discussion of our review. 

## 3. Results

### 3.1. Background and Definition

The very first description of this tumor was made by Beadles back in 1894 and it was considered a type of rodent ulcer [1,2]. He described a lesion with features both of BCC and SCC which could not be clearly separated [1,2]. Again, in 1910, in a larger series of rodent ulcers, McCormack wrote about tumors with intermixed basaloid and squamous features [2,3]. For the first time, in 1928, the term BSC was used by Montgomery in order to describe 17 out of 119 carcinomas which he believed were transitional between basal and squamous cell carcinomas [4,5]. 

In the following years, the origin and the definition of this entity troubled pathologists [6,7]. Some of them considered these lesions as collisions of separate primary BCC and SCCs, or variants of BCC that form keratin while others believed them to be independent tumors with features of both BCC and SCC [2,8,9]. Meanwhile, in part of the literature, BSC were referred to as “metatypical carcinomas” [5,10,11].

In more recent years, the theory of summarization is gaining ground [12]. According to this, the BSC is derived from a BCC with genetic alterations that undergo squamous differentiation [7,11,12]. This theory is reflected in the most recent definition of BSC by World Health Organization (WHO) in the textbook “WHO classification of skin tumors” which stated that: “Basosquamous carcinoma is a term used to describe basal cell carcinomas that are associated with squamous differentiation” [13]. At the same time, the National Comprehensive Cancer Network (NCCN) states that basosquamous carcinomas have a metastatic capacity that is more similar to that of SCC than BCC [5,7,14].

### 3.2. Epidemiology

BCCs and SCCs are the most common non-melanoma skin cancers with a rising incidence in the general population, while BSC is considered a relatively rare entity [7]. Up to date, very few small studies have evaluated the specific epidemiologic characteristics of BSC [5,6,15]. According to previous review papers, the incidence of BSC ranges from 1.7 to 2.7% [5].

Indeed, Shuller et al. reported a BSC incidence of 1.2%, Martin et al. reported a BSC incidence of 1.4% while Bowman et al.—in a retrospective study of cases treated with Moh’s micrographic surgery—found an incidence of 2.7% [6,16,17]. A newer retrospective study by Ciążyńska et al. revealed 180 lesions of BSC during a period of 20 years (1999–2019) which corresponded to 2.1% of all non-melanoma skin cancers (NMSCs) [7]. However, Gualdi et al., in a prospective study (2012–2015) which included 6042 NMSCs, reported a rate of 4.8% for BSCs, a percentage considerably higher than ever before [15].

### 3.3. Clinical and Demographic Characteristics

**Figure 1:**
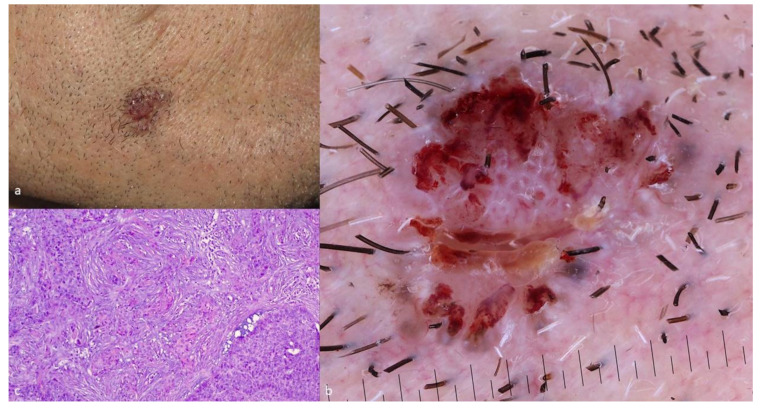
(**a**) Clinical image of a BSC; (**b**) dermoscopic image of a BSC revealing both a blue-grey ovoid nest and white circles; and (**c**) histologic image of a BSC (hematoxylin-eosin, 10×).

The clinical presentation of BSC is nonspecific and it does not present particular differences as compared to a common BCC (Figure 1a) [5,7]. The most common clinical scenario in BSC is a long-standing nodule that gradually becomes ulcerated. A similar clinical course was also described for metastatic BCCs [2,7,18]. Sun-exposed areas of the head and neck (82–97%), especially the perinasal area and ears, are the most frequent anatomic locations of this tumor [2,5,6,7,17,18,19]. However, though relatively fewer, BSCs were also observed in the trunk and extremities [2,5,6,7,17,18,19].

Fitzpatrick type I–II and high UVR exposure are considered potent risk factors for the development of a BSC [11]. The tumor usually develops in elderly individuals (34.4% of all BSCs are found in patients over 70 years of age) with a strong male preponderance [6,12,15,17].

### 3.4. Diagnosis of Basosquamous Carcinoma 

The early and concise diagnosis of this tumor is crucial due to its potentially aggressive biologic behavior. However, it is a rather intriguing task because there is an absence of a standardized diagnosing protocol. The differential diagnosis may include entities such as viral wart, seborrheic keratosis (SK), hyperkeratotic actinic keratosis (AK), Bowen’s disease (BD), BCC, invasive SCC, as well as amelanotic/hypomelanotic melanoma (AHM) [6,17,20,21]. 

#### 3.4.1. Dermoscopy of BSC

Dermoscopy reveals features unrecognizable during naked-eye clinical examination and it is recommended for the early recognition of skin cancer. In this context, dermoscopy may raise the suspicion of BSC by the recognition of certain dermoscopic characteristics, seen in both types of differentiation, namely basaloid and squamoid (Figure 1b). 

Unfortunately, due to the rarity of this entity, very few studies evaluating the dermoscopic features of BSC have been conducted until now [22,23]. In a study by Giacomel et al., the dermoscopic pattern of BSCs was characterized by features of both BCCs and SCCs, mirroring its complicated mixed histopathology [22]. In detail, the commonest dermoscopic criteria for BSCs were unfocused arborizing vessels, keratin masses, white structureless areas, scale, ulceration or blood crusts, white structures, blue-grey blotches and blood spots in keratin masses [22]. Finally, according to the authors, the most important dermoscopic clue that should raise suspicion for a possible BSC diagnosis is the simultaneous presence of at least one feature of both invasive SCC and BCC [22]. The study of Acay et al., in 2017, also demonstrated that BSC is dermoscopically characterized by BCC-related polymorphous or monomorphous vasculature, combined with findings of keratinization [23]. Specifically, a serpentine of branched vessels was the most common vascular finding while keratin masses, ulceration, and white structureless areas were the most common non-vascular features [23].

#### 3.4.2. Histopathologic Features of BSC

Biopsy and histologic examination remain the gold standard diagnostic method for BSC (Figure 1c). The majority of the published literature on the subject, mostly including case series, retrospective studies, and review articles, describe the presence of both BCC and SCC histologic characteristics with a transition zone between them. However, there is a certain controversy regarding how these features are arranged within the lesions [2,5,11]. The transition zone is considered, by most authors, as a tissue which depicts a transitional stage of differentiation between BCC and SCC cells and not simply an area with atypical BCC cells [2,5,24]. The BCC component of a BSC usually contains basaloid cells with a small cytoplasm and large, uniform, pale, nuclei, whilst the SCC element consists of accumulations of polygonal squamous cells containing voluminous eosinophilic cytoplasm, larger open nuclei with prominent nucleoli and frequent mitosis [2,17,24,25]. These aggregates of squamoid cells are either found inside the basaloid islands or as the other authors describe, adjacent to them [2,5,23,24,26,27]. Two entities that may mimic and should be considered in the histologic differential diagnosis of BSC are a collision tumor and a keratinizing BCC. In the former case, the BCC and SCC areas are completely separated with no transition zone and in the latter case, there is abrupt keratinization in the center of a nodular BCC lesion without the intervening areas of squamoid cells [1,2,28]. Finally, the correct histologic diagnosis of a BSC can be jeopardized when the biopsy is superficial and not incisional. In this scenario, the lack of deep areas of the lesion in the sample, where the squamoid characteristics often lay, may result in the incorrect interpretation of the tumor as a classic BCC [1,2].

#### 3.4.3. Immunohistologic Features of BSC

The histologic diagnosis of a BSC may be strengthened by the application of an immunohistochemical criterion such as human epithelial antigen (HEA) expression [20]. Despite the fact that no specific immunohistologic marker for BSCs exists, the Ber-EP4, an anti-HEA mouse monoclonal antibody, which is usually strongly positive in BCCs and the epithelial membrane antigen (EMA), which stains positive in SCCs, have proved to be helpful [2]. In a “typical” BSC, the BCC element is Ber-EP4, cytokeratin (AE1) and cytokeratin (AE3) positive, whereas the area of SCC is AE1, AE3, and CAM5.2 positive with variable staining for EMA [2,5,18]. Moreover, the transition zone, though not always present, is typically characterized by a gradual decline in Ber-EP4 staining as an indication of transitioning from the area of the basaloid to the area of squamous differentiation [2,5,29].

### 3.5. Genetics and Pathogenesis

The genetic origin of BSCs has not been fully clarified yet [5]. Although these tumors share histologic features of both BCCs and SCCs, the exact gene mutations that lead to their formation is still a matter of controversy [5,30]. On the other hand, the molecular background of BCC and SCC has been thoroughly studied. BCC derives from the over activation of the sonic hedgehog (HH) pathway which inhibits a transmembrane protein called PTCH or activates a transmembrane protein called SMO [31,32]. Other genetic drivers of BCC include PTEN, MYCN, PPP6C, GRIN2A, GLI1, CSMD3, DCC, PREX2, and APC [30,33,34]. SCC is characterized by a greater variety of gene mutations which include mutations in HRAS and disruptions of the TGFBR1, TGFBR2, NOTCH1, and NOTCH2 genes as well as additional mutations in CASP8, CDKN2A, NOTCH3, KRAS, NRAS, PDK1, BAP1, AJUBA, KMT2D, MYH9, TRAF3, NSD1, CDH1, and TP63 [30,35,36,37,38]. A recent, very interesting study by Chiang tried to define the genomic alterations that characterize BSC by using the targeted sequencing of 1641 cancer genes from 20 BSCs, whole exome sequencing from 16 BCCs, and a mixture of previously published whole-exome and whole-genome datasets from 52 SCCs [30,31,32,33,34,35,36,37,38,39]. According to the findings of this study, the majority of BSCs had underlying PTCH1 and SMO mutations in addition to mutations in other known BCC drivers such as MYCN, PPP6C, GRIN2A, CSMD3, DCC, PREX2, APC, PTEN, and PIK3CA [30]. These data support the theory that the HH signaling pathway is the initial driver of BSC and that this tumor probably originates as a BCC that partially squamatizes through the accumulation of ARID1A mutations and RAS/MAPK pathway activation [30,39]. The most frequent gene mutations that characterize BCCs, SCCs, and BSCs are shown in Table 1.

### 3.6. Biologic Behavior and Prognosis

According to many authors, in opposition to BCC, BSC is characterized by a more aggressive biologic behavior that is almost equal to that of SCC [2,5,18,25,40]. This “aggressiveness” is defined as a more dynamic topical growth of the tumor, as well as a greater potential for recurrences and metastases. As a consequence, the latter characteristics are associated with a worse prognosis as compared to the classic BCC [2,5]. 

Indeed, a relatively recent study by Zhu et al. including 19 patients with metastatic BSCs suggested an intermediate prognosis between that of BCC and SCC but more favorable than previous assumptions [40]. Volkestein et al. reported that the local recurrence rate of BSC—after wide surgical excision—may reach 45%, which is almost double that of BCC and SCC [18]. In another study, in which the physicians applied Mohs’ micrographic surgery, the topical recurrence rate for BSC decreased to 4–9%, but remained higher than BCC (0.64%) and SCC (1.2%) [2,14,41,42]. Martin et al. concluded that based on their analysis, the most important predictive factors of topical recurrence for BSCs were male sex, positive surgical margins, lymphatic, and perineural invasion [17]. The metastatic potential of BSC ranges between 4 and 8.4%, which is closer to that of SCC [2,41]. A recent study by Ciążyńska et al. reported that 40% of patients diagnosed with BSC had a second skin neoplasm. This percentage is significantly higher to the corresponding 23% reported with other non-melanoma skin cancers (NMSCs) and multiple lesions [7]. In this context, they concluded that BSC patients are more prone to the development of new primary skin cancers and for this reason, they should be closely monitored [7].

### 3.7. Therapeutic Options and Management of BSC

To date, there are no established, standard therapeutic guidelines for the treatment of BSCs. The rarity of these tumors along with the absence of robust literature data on the subject is the most reasonable explanation for this situation. Nevertheless, several treatment options have been applied with a variety of outcomes. Superficial methods such as curettage and electrodesiccation have been used in the past but are not considered first line treatment due to their high recurrence rate. 

#### 3.7.1. Wide Surgical Excision

Although a very high recurrence rate, reaching 45%, has been reported after wide surgical excision, this method remains a first line treatment choice for many authors [2,5,12,16,17,18,20,21,24,43]. Furthermore, it is suggested that surgical excision should be followed by the evaluation of a lymph node and distant metastases, and of course, close clinical follow up for recurrence and metastasis [2,6,17].

#### 3.7.2. Mohs’ Micrographic Surgery (MMS)

Based on the results of recent studies, MMS is considered the optimal surgical option for BSCs, since it is linked to lower recurrence rates compared to the wide surgical excision [17,24,25,44,45]. Analytically, Skaria et al. reported an 8.9% recurrence rate with MMS that is much lower than the 45% observed with wide surgical excision, but significantly higher when compared with the recurrence rates reported for BCCs and SCCs [44]. Allen et al. achieved an even lower recurrence rate (4.9%) [45]. However, according to Oldbury et al., there are several practical and financial issues to be addressed in order to officially support MMS as the first-line treatment of choice for BSCs: (a) inability to pre-operatively choose the right candidates for MMS (i.e., clinical diagnosis of BSC instead of BCC) in everyday clinical practice; (b) higher cost of MMS compared to surgical excision; (c) more time-consuming process; and (d) non-applicable in many centers worldwide [12]. 

#### 3.7.3. Sentinel Lymph Node Biopsy (SLNB)

SLNB has been suggested by some authors as part of the management of BSC. However, there is open controversy regarding whether SLNB should be offered in BSCs for the early diagnosis of occult nodal metastasis, staging and treatment of subclinical node disease [17,27]. Kakagia et al., in a prospective study with 142 patients with BSC, concluded that tumor size >2 cm in addition to lymphatic and perineural invasion are significant determinants of SLN micrometastasis [46]. In the absence of palpable lymphadenopathy, wide resection and SLNB with long-term follow up is highly recommended in these patients [46]. However, a possible “preventive benefit” of early SLNB for the nodal spread and distant metastasis needs further prospective controlled studies, with a longer follow-up period, in order to be confirmed [46].

#### 3.7.4. Radiotherapy–Chemotherapy

Adjuvant radiotherapy has been proposed by several authors for BSC, in the scenario of positive surgical margins and the inability to re-excise the tumor in order to achieve them, or in cases with local lymph node metastasis [2,12,17,47]. Although there are currently no reports in the literature about the use of radiotherapy in BSCs, some authors, based on their experience with aggressive and/or metastatic BCCs, suggest that treatment with radiotherapy either alone or in combination with surgery may be an appropriate option for the management of BSC, if standard surgical excision or MMS is not possible [12,48]. In a few cases of metastatic BSCs in the literature, palliative chemotherapy has been used (adriamycine, cisplatine) [11].

#### 3.7.5. New Emerging Therapies

Vismodegib, a Hedgehog pathway inhibitor, is approved for the treatment of adults with metastatic BCC, or with locally advanced BCC that has recurred after surgery, or adults who are not candidates for surgery or radiation [31]. According to the available literature data, the risk of SCC development in individuals treated with vismodegib is a matter of controversy [49,50,51]. Taking into consideration the fact that BSC pathologically displays features of both BCC and SCC, treatment with vismodegib might bear the risk of the progression of the SCC component of the tumor. Indeed, reports of SCCs developing in patients under vismodegib have been published and a case–control study with 180 patients found an increased risk of SCC in patients under treatment with vismodegib [49]. On the other hand, the largest published cohort study with 1675 patients suggested that vismodegib is not associated with an increased risk of SCC while a systematic and a narrative review of the literature concluded that the existing evidence does not justify an association between vismodegib and SCC formation [31,51,52]. Moreover, there are three very recent case reports including four patients in total which provide preliminary evidence that vismodegib might be effective for difficult-to-treat BSC (Table 2) [53,54,55]. In all of them, the lesions did not “transform” into purely squamous tumors but in contrast they completely remitted and in two cases the remission was maintained for a very long period of time [53,54,55]. Although the effect of vismodegib on BSC treatment definitely requires further investigation in larger controlled studies, it could prove to be a future solution for locally advanced BSCs despite the presence of a squamous component in the tumors.

Other treatments options such as the PD-L1 inhibitors pebrolizumab and cepilumab have been used for the treatment of advanced-metastatic BCCs and SCCs but still there are no literature data for their use in advanced BSC [56]. 

## 4. Conclusions

BSC remains a controversial entity, which belongs to the group of NMSCs, sharing characteristics of both BCC and SCC. The differential diagnosis between a BSC and a BCC cannot be made on the grounds of clinical examination. On the other hand, dermoscopy could be more helpful in the diagnosis by revealing features of both components (BCC, SCC) that are otherwise unrecognizable to the naked eye. Histology is the gold standard of diagnosis. Early recognition and correct histologic classification contribute to the optimization of the management of the tumor. Deep incisional biopsies and immunohistochemical techniques (Ber-EP4 staining) facilitate correct diagnosis. In the absence of standardized treatment protocols for BSC, prospective studies comparing various treatment options are needed, in order to reach a consensus regarding ideal management. Surgical treatments, including wide excision and Mohs’ micrographic surgery, remain the treatments of choice for most clinicians. The addition of SLNB, radiation therapy, and imaging monitoring in suspicious cases (tumor size >2 cm, perineural and lymphatic invasion) remain matters of controversy. Radiotherapy could have a supportive role, postoperatively, when re-excision is not possible or not allowed by the patient. Finally, a new treatment prospective such as vismodegib must be thoroughly investigated, with large controlled trials, since it may offer an alternative solution to irresectable or difficult-to-treat locally advanced cases of BSC.

## Figures and Tables

**Table 1:** The most frequent gene mutations that characterize BCCs, SCCs and BSCs.

BCCs	SCCs	BSCs
PTCH	HRAS	PTCH
SMO	TGFBR1	SMO
PTEN	TGFBR2	MYCN
MYCN	NOTCH1	PPP6C
PPP6C	NOTCH2	GRIN2A
GRIN2A	CASP8	CSMD3
GLI1	CDKN2A	DCC
CSMD3	NOTCH3	PREX2
DCC	KRAS	APC
PREX2	NRAS	PTEN
APC	PDK1	PIK3CA
	BAP1	ARIDIA
	AJUBA	
	KMT2D	
	CDH1	

**Table 2:** Studies which showed a complete response of locally advanced basosquamous carcinoma with vismodegib.

Source	Study Design	Number of Patients	Results
McGrane J. et al. [53]	Case report	1	Marked improvement, stable after 28 months
Apalla Z. et al. [54]	Case series	2	Complete clinical response with long-term follow up (12 and 18 months, respectively
Sahuquillo-Torralba A. et al. [55]	Case report	1	Complete response after 7 months
